# Cell Trajectory-Related Genes of Lung Adenocarcinoma Predict Tumor Immune Microenvironment and Prognosis of Patients

**DOI:** 10.3389/fonc.2022.911401

**Published:** 2022-07-18

**Authors:** Yu Luo, Xiaheng Deng, Jun Que, Zhihua Li, Weiping Xie, Guanqun Dai, Liang Chen, Hong Wang

**Affiliations:** ^1^ Department of Thoracic Surgery, The First Affiliated Hospital with Nanjing Medical University (Jiangsu Province Hospital), Nanjing, China; ^2^ Department of Respiratory and Critical Care Medicine, The First Affiliated Hospital with Nanjing Medical University (Jiangsu Province Hospital), Nanjing, China; ^3^ Department of General Practice, The First Affiliated Hospital with Nanjing Medical University (Jiangsu Province Hospital), Nanjing, China

**Keywords:** cell differentiation related genes (DRGs), immune microenvironment, prognosis prediction, lung adenocarcinoma, respiratory, WFDC2

## Abstract

**Background:**

Lung adenocarcinoma (LUAD) is the most common subtype of lung cancer which typically exhibits a diverse progression trajectory. Our study sought to explore the cell differentiation trajectory of LUAD and its clinical relevance.

**Methods:**

Utilizing a single-cell RNA-sequencing dataset (GSE117570), we identified LUAD cells of distinct differential status along with differentiation-related genes (DRGs). DRGs were applied to the analysis of bulk-tissue RNA-sequencing dataset (GSE72094) to classify tumors into different subtypes, whose clinical relevance was further analyzed. DRGs were also applied to gene co-expression network analysis (WGCNA) using another bulk-tissue RNA-sequencing dataset (TCGA-LUAD). Genes from modules that demonstrated a significant correlation with clinical traits and were differentially expressed between normal tissue and tumors were identified. Among these, genes with significant prognostic relevance were used for the development of a prognostic nomogram, which was tested on TCGA-LUAD dataset and validated in GSE72094. Finally, CCK-8, EdU, cell apoptosis, cell colony formation, and Transwell assays were used to verify the functions of the identified genes.

**Results:**

Four clusters of cells with distinct differentiation status were characterized, whose DRGs were predominantly correlated with pathways of immune regulation. Based on DRGs, tumors could be clustered into four subtypes associated with distinct immune microenvironment and clinical outcomes. DRGs were categorized into four modules. A total of nine DRGs (*SFTPB*, *WFDC2*, *HLA-DPA1*, *TIMP1*, *MS4A7*, *HLA-DQA1*, *VCAN*, *KRT8*, and *FABP5*) with most significant survival-predicting power were integrated to develop a prognostic model, which outperformed the traditional parameters in predicting clinical outcomes. Finally, we verified that knockdown of WFDC2 inhibited proliferation, migration, and invasion but promoted the apoptosis of A549 cells *in vitro*.

**Conclusion:**

The cellular composition and cellular differentiation status of tumor mass can predict the clinical outcomes of LUAD patients. It also plays an important role in shaping the tumor immune microenvironment.

## Introduction

Globally, non-small cell lung cancer (NSCLC) is among the most prevalent malignant diseases and is associated with high mortality (1). Lung adenocarcinoma (LUAD) is one of the most common subtypes of NSCLC. LUAD is a heterogeneous disease in terms of molecular alteration, pathological growth patterns, and clinical outcomes (2–4). There is considerable variability with respect to clinical outcomes even among patients with the same disease stage and treated with a similar therapeutic regimen. Surgical treatment is the only radical treatment for patients with early-stage LUAD; however, a proportion of patients tend to develop recurrence after tumor resection (5). Molecular alterations such as oncogenic mutations (*EGFR*, *KRAS*, *STK11*) usually help to define certain subtypes of lung cancer that share similar biological features (6–8). However, tumors with similar molecular alteration may still exhibit differences in terms of tumor aggressiveness and therapeutic response (9–12). In addition to targeted therapy and conventional chemotherapy, immunotherapy is a milestone therapeutic regimen for lung cancer which is widely used for the treatment of advanced LUAD (13, 14). However, the therapeutic efficacy of immunotherapy shows considerable interindividual variability, with some patients achieving a durable response, while others showing no response at all (15). The underlying mechanisms responsible for the distinct aggressiveness and therapeutic response in LUAD are yet to be elucidated.

Tumor mass is a heterogeneous entity composed of stromal cells and malignant cells with variable differentiation status (16). Cellular differentiation status plays a critical role in tumor development and progression (16). Tumors predominantly comprising poorly differentiated cells with stem cell features were shown to be associated with rapid progression and unfavorable outcomes (17). The differentiation status of tumor cells can dynamically change during the progression of malignant disease; a typical example of this phenomenon is the epithelial–mesenchymal transition (EMT) which is characterized by the acquisition of a mesenchymal-like phenotype by epithelial cells (18). EMT is associated with a more aggressive phenotype, greater propensity for metastasis, and resistance to all kinds of antitumor therapy (19, 20). The tumor immune microenvironment can also be remodeled by the differentiation status of tumor cells, as stem cell property and EMT were shown to be associated with an immune suppressive phenotype (21). The cellular composition of tumor mass also plays a role in determining the biological features of the tumor. The immune context, such as the infiltration level and the functional status of different immune cells within the tumor mass, is closely associated with cancer prognosis and therapeutic response (22, 23). Certain stromal components in the tumor mass such as the infiltrating or resident myeloid cells also demonstrate high plasticity, as their phenotype dynamically changes along with tumor progression and helps modulate the tumor microenvironment and disease progressiveness (24). To summarize, the cellular composition and differentiation status of intratumor cells can play a key role in determining tumor biological features, microenvironment, and clinical outcomes.

The traditional approach to study tumor cell heterogeneity relies on some known biomarkers, which is less effective and provides limited information. The development of the whole-exome RNA-sequencing technique and single-cell RNA-sequencing (scRNA-seq) technique has provided new tools for cancer study. scRNA-seq is a powerful tool to explore intra-tumor heterogeneity and biological behaviors. Publicly available databases such as The Cancer Genome Atlas (TCGA) and the Gene Expression Omnibus (GEO) also facilitate cancer research with abundant RNA-sequencing data as well as complete clinical information. In this study, we utilized three independent datasets of adenocarcinoma (one scRNA-sequencing dataset and two bulk-tissue RNA-sequencing datasets), to explore the cellular composition and differentiation trajectory of intratumor cells, and assessed their clinical implications. We first classified intratumor cells into different branches with distinct expression patterns based on trajectory analysis using the scRNA-seq dataset. The feature genes that were highly expressed in each branch were defined as differentiation-related genes (DRGs), whose clinical relevance was evaluated through the RNA-sequencing data of bulk tumors. Further, we developed a prognostic nomogram integrating DRGs with prognostic significance and clinicopathological features and validated it in an independent dataset. Finally, the functions of WFDC2 were verified in A549 cells. Our findings highlight the importance of intratumor cellular composition and differentiation status in remodeling the tumor immune microenvironment and predicting clinical outcomes.

## Materials and Methods

### Data Acquisition and Preprocessing

One scRNA-sequencing dataset (GSE117570) from the GEO database and two bulk-tissue RNA-sequencing datasets (TCGA_LUAD dataset from TCGA and GSE72094 dataset from GEO) were included in this study. Tumor bulk-tissue RNA-sequencing data and the corresponding clinical information of 594 patients with LUAD were obtained from TCGA database (https://portal.gdc.cancer.gov/) and used as the training cohort. The GSE72094 dataset containing RNA-sequencing and clinical data of 442 patients with LUAD were also collected as the validation dataset (25).

GSE117570 contains single-cell sequencing data of dissociated tumors or adjacent normal tissue from four NSCLC patients (three lung adenocarcinoma and one squamous cell lung cancer) (24). After exclusion of data from squamous cell lung cancer and its corresponding normal tissue, sequencing data from a total of 3,183 cells derived from three adenocarcinomas and their adjacent normal tissues were subjected to quality check and further study, which were all carried out by the Seurat package in R environment (R 3.5.1) (26). The percentage of mitochondrial genes was calculated by the PercentageFeatureSet function. All sequencing data had a reading depth of 10× genomics based on Illumina NextSeq 500. The relationship between sequencing depth and mitochondrial gene sequences, as well as total intracellular sequences, was evaluated by correlation analysis. HTseq counts of the remaining cells were normalized using a linear regression model with the LogNormalize method. During quality control, we further excluded a total of 2,078 low-quality cells with genes detected in <3 cells, <50 total detected genes, or ≥5% of mitochondria-expressed genes. The remaining cells were normalized using a linear regression model with the LogNormalize method. The top highly variant 1,500 genes were also identified by variance analysis.

Cell annotation by dimensionality reduction and trajectory analysis with pseudotime PCA was performed to identify significantly available dimensions with a p-value < 0.05 (27). Cluster classification analysis with dimensionality reduction for the top 15 principal components (PCs) was carried out using the t-distributed stochastic neighbor embedding (tSNE) algorithm to obtain the major clusters (28). Marker genes of each cluster were identified by differential expression analysis conducted using the limma package in R environment (29). Genes with an adjusted *p* value < 0.05 and | log2[fold change (FC)] | > 0.5 were considered to be significant. Annotation of cell clusters was carried out by the singleR package according to the composition patterns of the marker genes and were then manually verified and corrected with the CellMarker database (30, 31).

We then constructed the single-cell pseudotime trajectory using the Monocle 2 algorithm based on the scRNA-seq data derived from lung adenocarcinoma (32). We can interpret the differentiation trajectory of every single cell, as they were projected onto this space and ordered into a trajectory with branch points; cells within the same branch were considered to have a similar differentiation state, and vice versa. We also identified differentially expressed genes in cells with distinct differentiation states with | log2[fold change (FC)] | > 0.5 and FDR <0.05, which were defined as state-specific marker genes and were also defined as DRGs in our study. DRGs of different branches were applied to Gene Ontology (GO) and Kyoto Encyclopedia of Genes and Genomes (KEGG) analyses to identify the enriched pathways. All pathway analyses were implemented by the “clusterProfiler” package in R environment. Pathways with an adjusted p value < 0.05 were considered to be significantly enriched.

### Classification of LUAD Patients Based on DRGs in the GSE72094 Dataset

To employ the DRGs identified from the trajectory analysis for classification of LUAD patients based on the bulk transcriptome data, we performed unsupervised consensus clustering on the GSE72094 dataset using the above-identified DRGs. Unsupervised consensus clustering was implemented by ConsensusClusterPlus’ package in R, using an algorithm based on k-means machine learning (33). The optimal number of clusters was decided based on the relative change in the area under the cumulative distribution function (CDF) curves of the consensus score and consensus heatmap. The clinical relevance of the clustering was evaluated in terms of its correlation with clinical features such as overall survival, age, sex, tumor stage, and oncogene mutation.

### Tumor Purity Prediction and Enumeration of Immune Cells

Tumor purity and the presence of infiltrating stromal/immune cells in tumor tissues were inferred based on the expression data using an ESTAMATE algorithm. Single sample gene set enrichment analysis (ssGSEA) and ESTAMATE signatures were integrated in the ESTAMATE algorithm, whose outputs include stromal score (represents the presence of stroma in tumor tissue), immune score (represents the infiltration of immune cells in tumor tissue), and estimate score (represents tumor purity) (34).

The composition of different immune cells was inferred by CIBERSORT, which is a web portal (http://cibersort.stanford.edu/) that performs cell type enrichment analysis for different immune cell types based on the bulk-tissue RNA-seq data (35). The relative infiltrating fractions of the 22 immune cell types in the tumor microenvironment were estimated by CIBERSORT.

### WGCNA Analysis

Utilizing TCGA dataset, we conducted weighted gene co-expression network analysis (WGCNA) to explore the co-expression network of all the DRGs, which was implemented by the WGCNA package in R software. The distance between each DRG was calculated using the Pearson correlation coefficient, and a weighted co-expression network was constructed. Genes with missing values were identified and discarded, and a cluster tree was constructed to test the outliers. Network topology analysis was then applied to choose the soft-thresholding power. The expression matrix was transformed into an adjacency matrix and, subsequently, into a topological matrix (TOM). The hierarchical clustering tree of genes was shaped with the corresponding dissimilarity calculated. Based on the TOM, we used the average-linkage hierarchical clustering method to cluster genes. Modules with similar patterns through the Dynamic Tree Cut were identified and merged. We followed the standard of a mixed dynamic shear tree and set the minimum number of genes in each DRG network module to 20. Gene Significance (GS) and Module Membership (MM) were the quantifications of the relationship of genes to the trait and modules. Genes in the modules demonstrating the most remarkable association with clinical traits (such as age, sex, smoking history, stage, survival status, and survival time) were identified for further analysis. Genes with GS >0.5 and MM >0.5 were selected as candidate hub genes.

### Identification of Hub Genes and Construction of the Risk Prediction Model

The candidate hub genes identified in WGCNA analysis were further applied to univariate survival analysis. Genes with a Univariate Cox value of p < 0.05 were further screened with the least absolute shrinkage and selection operator (Lasso) Cox model, which was implemented by package glmnet in R environment (36). Genes screened by Lasso Cox regression analysis were selected as hub genes and used for the construction of the risk prediction model, which was denoted as RiskScore in our study. Lambda derived from Lasso analysis was used as the coefficient of each gene in the calculation of RiskScore with the following formula: RiskScore =


$$\begin{array}{l} \sum_{n}^{t}\left(Exp_{t}+Coef_{t}\right) \end{array}$$

where t represents the “t”th gene and n represents the total number of genes. The GSE72094 dataset was used as a testing cohort for external validation of RiskScore. The predictive power of the nomogram in the training cohort and validation cohort was evaluated and presented using receiver operating characteristic (ROC) curve analysis.

### Construction and Validation of the Prognostic Nomogram

TCGA_LUAD dataset was applied as a training set for the development of a prognostic nomogram. Overall survival (OS) was defined as the time from the date of diagnosis or surgical resection to the date of death or most recent follow-up. Clinical parameters such as age, TNM stage, and RiskScore were included in univariate and multivariate Cox regression analyses using the backward stepwise Cox proportional hazard model. The coefficients for each parameter derived from multivariate analysis were used for the construction of a prognostic nomogram. The survival statuses of patients at 1, 3, and 5 years were used as the endpoint parameters for the development of the nomogram (37). The discrimination and calibration of the nomogram for the endpoint index were measured by the concordance index (C-index = 0.727) and by calibration plot comparing the expected and observed survival probabilities, respectively. The GSE72094 dataset was used as a testing cohort for external validation. The predictive power of the nomogram in the training cohort and validation cohort was evaluated and presented using the ROC curve.

### Cell Culture

Human lung cancer cell lines (A549) were purchased from the American Type Culture Collection (ATCC, USA). Cells were cultured with Dulbecco’s Modified Eagle’s Medium/Nutrient Mixture F-12 (DMEM/F12) (DMEM/F-12; Invitrogen, Carlsbad, CA, USA) supplemented with 10% fetal bovine serum (FBS; Gibco, Grand Island, NY, USA) and 4 mM of L-glutamine. Cells were cultured and incubated in a 5% CO_2_ atmosphere at 37°C.

### siRNA Transfection

A549 cells (5 × 10^4^ cells per well) were seeded in 24-well plates and cultured overnight. Cells were transfected with 100 nM non-targeting siRNA, si-WFDC2 (Ruibo, Liaocheng, Shandong, China) using the Lipofectamine 3000 Transfection Reagent (Thermo Fisher, Waltham, MA, USA) following the manufacturer’s instructions. The negative control group was treated only with a transfection reagent. After 48 h, the transfection efficiency was confirmed by qRT-PCR.

### EdU Cell Proliferation Assay

The influence of WFDC2 on the proliferation of A549 cells was further evaluated using the EdU Apollo 567 *In Vitro* Kit (Solarbio, Beijing, China). Briefly, A549 cells transfected with or without si-WFDC2 were placed in 96-well plates at the density of 1 × 10^3^ cells. EdU solution (50 µM) was added to each well for 2 h. Then, cells were fixed with 4% paraformaldehyde for 10 min at room temperature. Cells were treated with glycine (2 mg/ml) and Apollo (1×), respectively. The nuclei were stained with 4′,6-diamidino-2-phenylindole (DAPI, Sigma, St. Louis, MO, USA). Positive staining was observed and captured under a fluorescence microscope (Olympus, Tokyo, Japan).

### CCK-8 Assay

The proliferation of A549 cells transfected with or without si-WFDC2 cells were analyzed using the Cell Counting Kit-8 assay. Cells were seeded at 1 × 10^3^ cells per well into 96-well plates and cultured overnight at 37°C and 5% CO_2_. After 24 h, CCK‐8 (Dojindo, Kumamoto, Japan) was added to each well and cultured for 2 h. The OD values at 450 nm were detected using the Multiskan Go Spectrophotometer (Thermo Fisher Scientific, USA) at days 1, 2, 3, and 4, respectively.

### Colony Formation Assay

A549 cells transfected with or without si-WFDC2 (1 × 10^3^) were first blended into top agar (1.5 ml) for colony formation assay. Then the mixture was added onto base agar in each well. Three weeks post‐seeding, colonies were stained with 0.5% Crystal Violet for 15 min. To count the colonies, a single-lens reflex camera (Nikon) was used.

### Apoptosis Assay

Cell apoptosis was evaluated utilizing FITC Annexin V Apoptosis Kit (BD Biosciences, San Jose, CA, USA) by flow cytometry assay. A549 cells (5 × 10^5^) transfected with or without si-WFDC2 were incubated with 1× binding buffer (100 μl) supplemented with PI (5 μl) and FITC Annexin V (5 μl) at room temperature and then analyzed using a flow cytometer (BD Biosciences).

### Cell Cycle Analysis

The cell cycle was analyzed by a Cell Cycle Detection Kit (Keygen Biotech, Nanjing, China). Briefly, A549 cells transfected with or without si-WFDC2 were collected and fixed in 70% cold ethanol overnight. After that, cells were treated with 100 μl RNase A (100 mg/ml) for 30 min at 37°C and stained with 400 μl PI (50 mg/ml) at 4°C for 30 min. DNA content was measured using flow cytometry (Beckman Coulter FC500); the number of cells in the G1, S, and G2 phases were quantified by cell-cycle analysis software (FlowJo v10.0, Palo Alto, CA, USA).

### Transwell Assay

The migration and invasion of A549 cells were evaluated using Transwell assays. A549 cells transfected with or without si-WFDC2 were pre-starved for 12 h, and 5 × 10^4^ cells were seeded in the upper chambers of the Transwell inserts coated with or without Matrigel^®^ (BD Biosciences, Bedford, MA, USA) solution, and the medium (DMEM supplemented with 10% FBS) was added into the lower chambers. After a 24-h incubation, the migrated and invaded cells were fixed with formalin for 10 min and stained using 0.1% crystal violet for 20 min at room temperature. Finally, the cell numbers were counted in five randomly selected fields under the microscope (Olympus, Japan).

### qRT-PCR

Total RNAs were extracted using TRIzol^®^ Reagent (Invitrogen; Thermo Fisher Scientific, Inc.) according to the manufacturer’s instructions. The RNAs were transcribed into cDNAs with First Strand cDNA Synthesis Kit (Thermo Scientific, USA). Specific cDNAs were amplified with iTaq™ Universal SYBR^®^ Green (Bio-Rad, Hercules, CA, USA) utilizing Eco Real-Time PCR System (Illumina, San Diego, CA, USA). The results were analyzed using the 2−ΔΔCT relative quantitative method, with GAPDH as an internal control.

### Statistical Analysis

Between-group differences with respect to continuous variables were assessed using two-sample *t* test or Wilcoxon test. Between-group differences with respect to categorical variables were assessed using the χ^2^ test, CMH-χ^2^ test, or Fisher’s exact test, as appropriate. Pearson correlation analysis was conducted to assess the correlation between two continuous variables. Continuous variables were transferred to dichotomous variables with the median value as the cutoff point before inclusion in survival analysis. Log-rank test was used to evaluate the survival relevance of different parameters, which were presented with Kaplan–Meier plots. Logistic regression univariate analysis was conducted to explore the prognostic significance of each DRG. ROC curve analysis was performed to assess the performance of RiskScore in predicting the postsurgery survival probability of LUAD at 1, 3, and 5 years, respectively. All statistical analyses and data presentations were performed in R language 3.5.1 (http://www. r-project.org). p values less than 0.05 were considered indicative of statistical significance.

## Results

### scRNA-Seq Analysis Identified 10 Clusters of LUAD Cells

**Figure 1:**
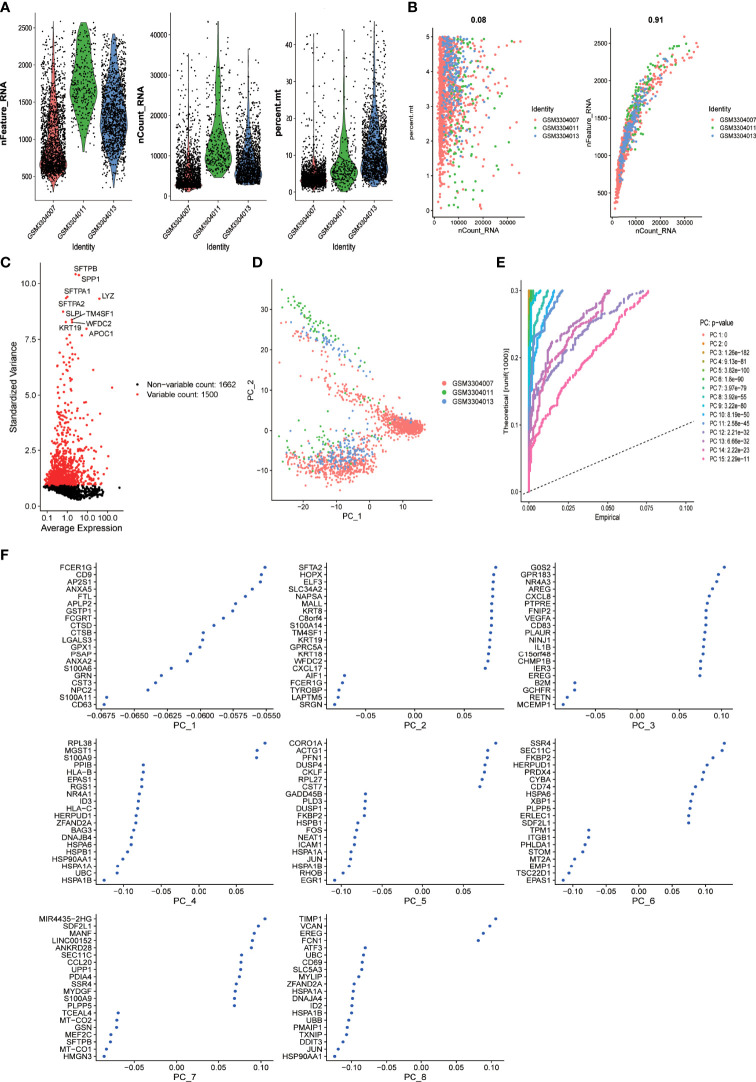
scRNA-seq analysis identified eight clusters of LUAD cells **(A)** Violin plots demonstrate the sequencing depth (left), number of detected genes (middle), and percentage of mitochondria genome (right) in each single-cell samples. After quality control of the 3,183 cells from the tumor cores of three human LUAD samples, 1105 cells were included in the analysis. **(B)** Dot plot demonstrates the correlation between sequencing depth and percentage of mitochondrial genes across all the single-cell samples (left). Dot plot demonstrates the correlation between sequencing depth and total intracellular sequences (right). **(C)** Dot plot demonstrates the correlation variance of each gene expression across all the single-cell samples. A total of 3,162 genes throughout all cells of LUAD are shown in the variance diagram. A total of 1,500 genes found to be highly variable are denoted as red dots, and 1,662 genes without significant variation are represented by black dots. Gene symbols of the top 10 highly variable genes are shown. **(D)** Principal component analysis (PCA) cannot clearly separate LUAD cells. **(E)** PCA identified 15 principal components (PCs) with an estimated P value less than 0.05. **(F)** The dimensionality of the 15 PCs were reduced using the tSNE algorithm and successfully yielded eight cell clusters whose top 20 markers genes are shown in dot plots.

Single-cell sequencing data from dataset GSE117570 were analyzed. Standard quality control and normalization were performed for a total of 5,189 cells derived from the tumor mass of three cases of lung adenocarcinoma and 3,183 cells derived from adjacent normal tissues. A total of 2,078 low-quality cells were excluded from further analysis (**Figure 1A**). Significant positive correlations between sequencing depth and mitochondrial gene sequences (R = 0.08) and between sequencing depth and total intracellular sequences (R = 0.91) were detected (**Figure 1B**). A total of 3,162 genes detected in the dataset were included in the study. Variance analysis was performed for all the included genes, among which 1,500 genes were found to be highly variable (p < 0.05 and log(fold change) >1) across cells, while 1,662 genes showed no significant variation (**Figure 1C**). To classify the cells derived from tumor mass into different subsets, principal component analysis (PCA) was performed based on scRNA sequencing data. Although a total of 15 principal components (PCs) were identified (**Figure 1E**), PCA could not clearly separate the cells (**Figure 1D**). The top 20 highly expressed genes that were highly correlated with each of the first eight components are shown in **Figure 1F**.

To further identify cell clusters with distinct features, the t-distributed stochastic neighbor embedding (tSNE) algorithm was used to narrow down the cells into 10 distinct clusters. We also performed differential expression analysis to identify differentially expressed genes in different clusters, which were defined as the marker genes of the clusters. A total of 1,187 marker genes were identified; the top 20 marker genes for each cluster are presented in the heatmap (**Supplementary Figure 1**).

### Trajectory Analysis Identified Four Branches of Cells From LUAD Tumor Mass and Their Corresponding DRGs

**Figure 2:**
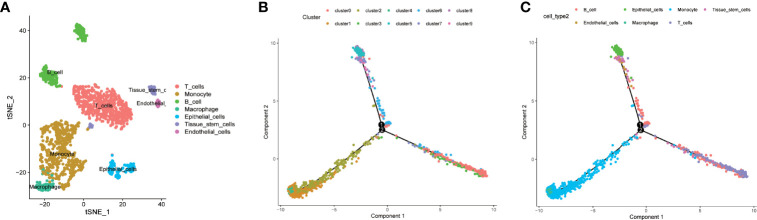
Cell annotation, differentiation trajectory prediction, and pathway enrichment analysis of different branches. **(A, B)** All 10 clusters of cells in LUAD were annotated by singleR and CellMarker according to the composition of the marker genes. **(C)** All cells from tumor mass with cellular annotation were projected into a three-dimensional graph with one root (II) and four branches (branches I, III, IV, and V) in the trajectory analysis. Four branches of cells (branches I, III, IV, and V) with distinct differentiation patterns were identified by trajectory analysis.

The 10 clusters identified previously were annotated using singleR and CellMarker. Based on the expression pattern of the marker genes, the cellular subsets that were recognized during the annotation included T cells (cluster 0, containing 566 cells), monocytes (clusters 1 and 2, containing 334 cells and 265 cells, respectively), B cells (clusters 3 and 6, containing 130 cells and 86 cells, respectively), macrophages (cluster 4, containing 89 cells), epithelial cells (clusters 5 and 8, containing 87 cells and 51 cells, respectively), tissue stem cells (cluster 7, containing 57 cells), and endothelial cells (cluster 9, containing 30 cells) (**Figure 2A** and **Supplementary Table 6**).

We further performed trajectory analysis to identify cells with a distinct differentiated pattern. All the cells derived from the tumor mass were projected into a three-dimensional graph with one root (II) and four branches (branches I, III, IV, and V) (**Figures 2B, C**). Branch-specific feature genes were identified as genes that were incrementally upregulated or downregulated from root to each branch (**Supplementary Tables 1–4**). Feature genes that were upregulated in each branch were defined as differentiation-related genes (DRFGs) and were applied to GO and KEGG pathway analyses; all the significantly enriched pathways for different branches are shown in **Supplementary Figures 2A–D**. Pathways that were significantly enriched in each branch were mainly associated with immune regulation, such as immune cell infiltration or activation, antigen presentation, and cytokine production. The DRFGs in branches I/V were involved in neutrophil degranulation and activation, immune receptor activity, and inflammatory disease occurrence (**Supplementary Figures 2A, D**); those in branch II were also associated with a positive regulation of cytokine production and antigen processing and presentation (**Supplementary Figure 2B**), and those in branch III were closely linked to response to molecule of bacterial origin and *Staphylococcus aureus* infection pathways (**Supplementary Figure 2C**).

### DRGs-Based Classification of LUAD Patients Correlated With Distinct Clinical Outcomes

**Figure 3:**
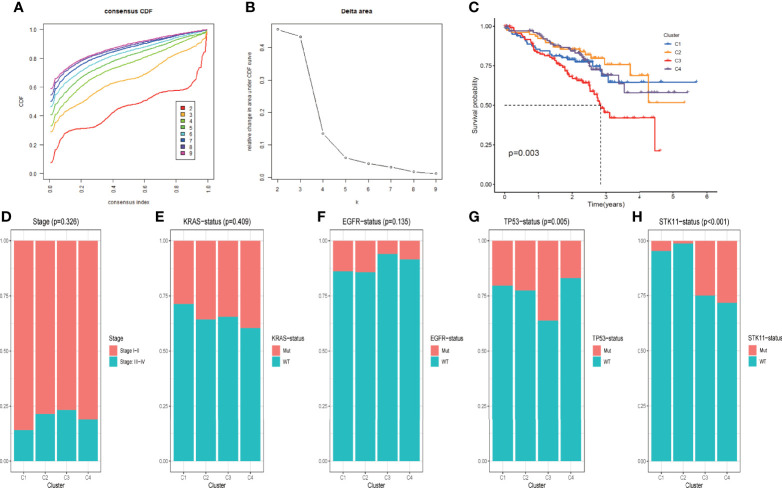
DRG-based classification of LUAD patients and their corresponding clinicopathological features **(A)** CDF curves of the consensus score (k = 2–9) in the GSE72094 cohort. **(B)** Relative change in the area under the CDF curve (k = 2–9) in the GSE72094 cohort. **(C)** Kaplan–Meier survival analyses of patients in clusters C1, C2, C3, and C4 in the GSE72094 cohort. **(D–H)**. Comparisons of the clinicopathological variables including stage **(D)**, *KRAS* mutation status **(E)**, *EGFR* mutation status **(F)**, *TP53* mutation status **(G)**, and *STK11* mutation status **(H)** among patients with clusters C1, C2, C3, and C4 in the GSE72094 cohort.

To classify LUAD patients based on the expression pattern of DRGs identified from scRNA sequencing analysis, machine learning-based unsupervised consensus clustering was carried out using the bulk transcriptome data of 442 LUAD tumors from the GSE72094 dataset. The number of clusters (k value) was determined based on the area under the CDF curve and consensus heatmap. When the k value was 4, the heatmap clearly classified the tumor into four clusters with distinct expression patterns (**Supplementary Figure 3A**), and the relative change in the area under the CDF curve was minimal with the increase in the k value (**Figures 3 A, B**). Thus, all LUAD patients from the GSE72094 dataset were classified into four groups, and their correlation with clinicopathological features was evaluated. The Kaplan–Meier survival plot was used to present the survival difference among patients of different groups; the results showed that patients of C3 had the worst overall survival compared with the other three groups (p = 0.003) (**Figure 3C**). The distribution of different clinicopathological features such as age, sex, smoking history, tumor stage, and mutation status of oncogenes or tumor-suppressor genes (*KRAS*, *EGFR*, *TP53*, *STK11*) in different groups was compared and presented as percentage bar plot (**Figures 3D–H**, **Supplementary Figures 3B–D**). The distribution of all these clinical features was comparable across the four groups except for the mutation status of *TP53* and *STK11*. *TP53* mutation occurred more frequently among patients of C3 (**Figure 3G**). The mutation of *STK11* was significantly more frequent in C3 and C4 and extremely rare in C1 and C2 (**Figure 3H**).

### DRG-Based Classification of LUAD Demonstrates Distinct Immune Microenvironment

**Figure 4:**
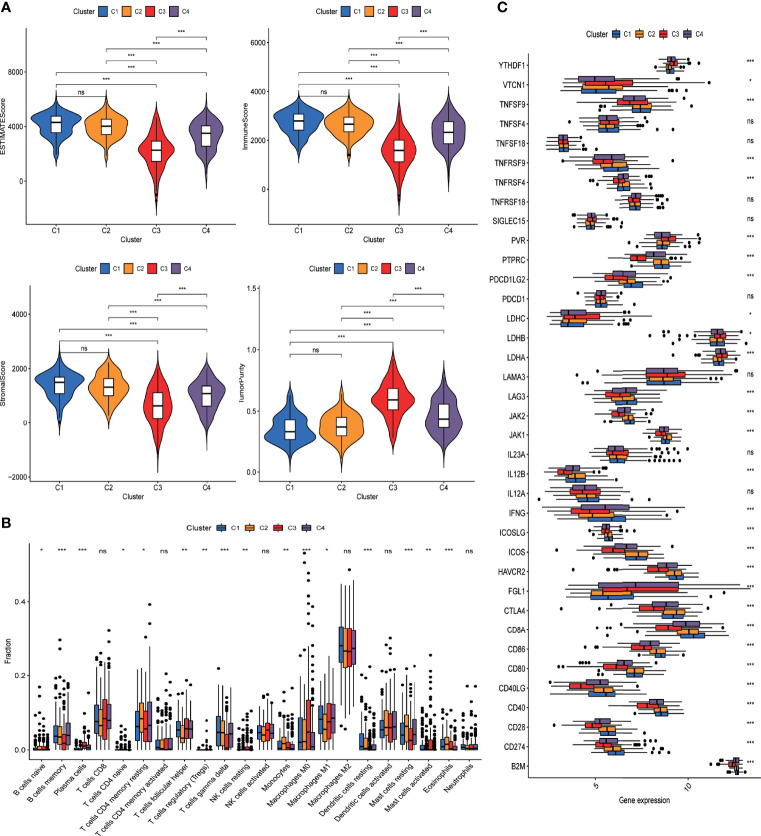
DRG-based classification of LUAD patients and their corresponding tumor immune microenvironment. **(A)** Violin plots present the ESTIMATEscore of tumors from C1, C2, C3, and C4 clusters in the GSE72094 cohort. Intercluster comparison was performed using *t* test. *p < 0.05; **p < 0.01; ***p < 0.001; ns, not significant. **(B)** Boxplot showing the infiltrating fractions of 22 different subtypes of immune cells in the four groups of tumors. Between-group differences were evaluated using the Wilcoxon test. *p < 0.05; **p < 0.01; ***p < 0.001; ns, not significant. **(C)** Boxplot showing the expression levels of immune check point genes across four groups of tumors. Between-group differences were evaluated using Wilcoxon test. *p < 0.05; **p < 0.01; ***p < 0.001; ns, not significant.

We further evaluated the difference in the tumor microenvironment of the four different groups. As shown in **Figure 4A**, tumors from different groups significantly differed from one another in terms of immune score, stromal score, and tumor purity as inferred from the expression data. Of note, tumors from C3 demonstrated the lowest immune score and stromal score but the highest score for tumor purity. Also, tumors of the four groups differed from each other with respect to the composition of different immune cells (**Figure 4B**, **Supplementary Table 7**). Specifically, tumors of C3 had a significantly lower level of adaptive immune population (such as B cells and T cells) but significantly more abundant innate immune cells (such as NK cells and macrophages) compared with other groups (**Supplementary Figure 3E**). We further evaluated the difference in immune checkpoint expression among tumors of the four groups. As shown in **Figure 4C**, the expression pattern of most of the known immune checkpoints was significantly different among the four groups.

Considering the significant difference in immune characteristics and overall survival among patients of the four groups, we anticipated that the immune microenvironment plays an important role in dictating tumor progression and clinical outcome. We further evaluated the prognostic relevance of different immune populations as well as immune-associated genes in LUAD patients using the GSE72094 dataset. As shown in **Supplementary Figure 1**, lower levels of macrophage M0, activated mast cells, and plasma cells and higher levels of resting mast cells and CD4 memory resting cells were associated with significantly prolonged overall survival (p < 0.05). Immune-associated genes whose overexpression predicted favorable survival included *CD40LG* and *PTPRC*. Some other immune-associated genes such as *YTHDF1*, *LDHA*, *PVR*, and *TNFSF4* correlated with unfavorable prognosis when highly expressed.

### RiskScore Calculated With Prognosis-Associated DRGs Outperformed Conventional Parameters in Predicting Clinical Outcomes

**Figure 5:**
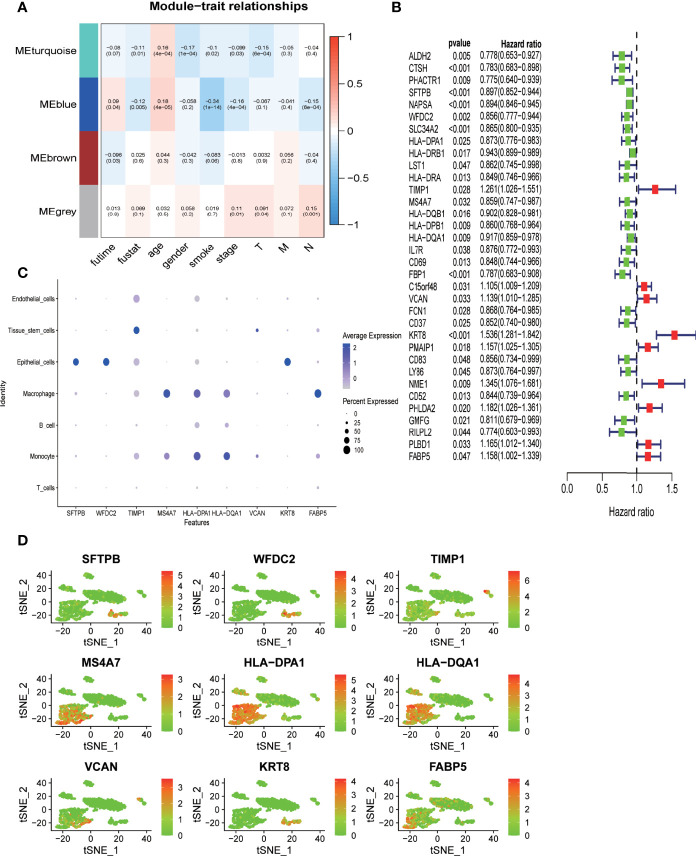
Deidentification of hub-DRGs for the prognostic model (RiskScore) **(A)** Heatmap showing the correlation between modules of DRGs derived from WGCNA analysis and clinical traits such as age, sex, smoking status, TNM stages, survival time, and survival status in TCGA-LUAD cohort. The correlation coefficient and p value (in parentheses) are shown. **(B)** Forest plot demonstrating DRGs with significant prognostic impact in TCGA-LUAD cohort. Genes were converted into dichotomous variable with median value as the cutoff level; subsequently, logistic regression univariate analysis was conducted to explore their correlation with overall survival. Genes associated with favorable survival are denoted in green in the forest plot, while those associated with unfavorable prognosis are denoted in red. **(C, D)**. Expression level of the nine hub genes across different cell types in nine clusters as classified by the tSNE algorithm.

To better classify DRGs based on their co-expression pattern, WGCNA was performed for DRGs that are specific to each branch to construct co-expression patterns using TCGA-LUAD dataset. A total of four modules, MEturquoise, MEblue, MEbrown, and MEgrey, were identified, containing 326, 43, 29, and 14 DRGs, respectively (**Supplementary Table 5**). The correlation between the expression of each module and clinical traits such as age, sex, smoking history, stage, survival status, and survival time was evaluated. As shown in **Figure 5A**, two modules (MEturquoise and Eblue) demonstrated the most remarkable association with clinical traits. Both MEturquoise and MEblue showed a significant correlation with deceased survival status, older age, no smoking history, and early tumor stage (**Figure 5A**). DRGs from modules MEturquoise and MEblue were selected as potential hub DRGs for the construction of the DRG-based prognostic model. All the DRGs from modules MEturquoise and MEblue were applied to differential expression analysis to identify their differential expression status between cells from the tumor mass and normal tissue (**Figure 5B**, **Supplementary Figure 5**). A total of 15 genes were highly expressed in cells derived from tumor mass, and 85 genes were highly expressed in cells from normal tissue; the rest of the DRGs demonstrated no differential expression (**Figure 5B**, **Supplementary Figure 5**).

Univariate Cox-regression survival analysis was performed to identify potential hub DRGs with significant prognostic relevance, which was further applied to multivariate Cox-regression survival analysis to evaluate their independent prognostic significance (**Figure 5B**). All survival analyses were based on TCGA-LUAD dataset. Only genes with independent prognostic significance were applied to the development of a prognostic model. The lambda value from Lasso analysis was carried out to construct the prognostic model.

A total of nine DRGs (*TIMP1*, *VCAN*, *HLA-DPA1*, *HLA-DQA1*, *KRT8*, *MS4A7*, *SFTPB*, *WFDC2*, *FABP5*) were finally recruited for the development of the prognostic model (Risk score) using TCGA-LUAD dataset. Pairwise comparison was carried out to evaluate the differential expression status of all the hub genes between tumor tissues and adjacent normal tissues using the expression data derived from 19 paired samples from our center (38). As shown in **Supplementary Figure 2**, *FABP5* and *MS4A7* demonstrated a significantly higher expression in normal tissue (p < 0.05), while the expression levels of *TIMP1*, *KRT8*, and *WFDC2* were significantly higher in tumor tissue (p < 0.05); the four remaining genes *VCAN*, *HLA-DPA1*, *HLA-DQA1*, and *SFTPB* demonstrated no significant differential expression between tumor and normal tissue (p > 0.05). The expression level of the hub genes in different cell types as annotated based on scRNA sequencing data was evaluated (**Figures 5C, D**). *SFTPB*, *WFDC2*, and *KRT8* were highly expressed in epithelial cells; *MS4A7*, *HLA-DPA1*, and *HLA-DQA1* were mainly expressed in immune cells such as macrophages and monocytes; and *TIMP1* and *VCAN* were predominantly expressed by tissue stem cells (**Figures 5C, D**).

**Figure 6:**
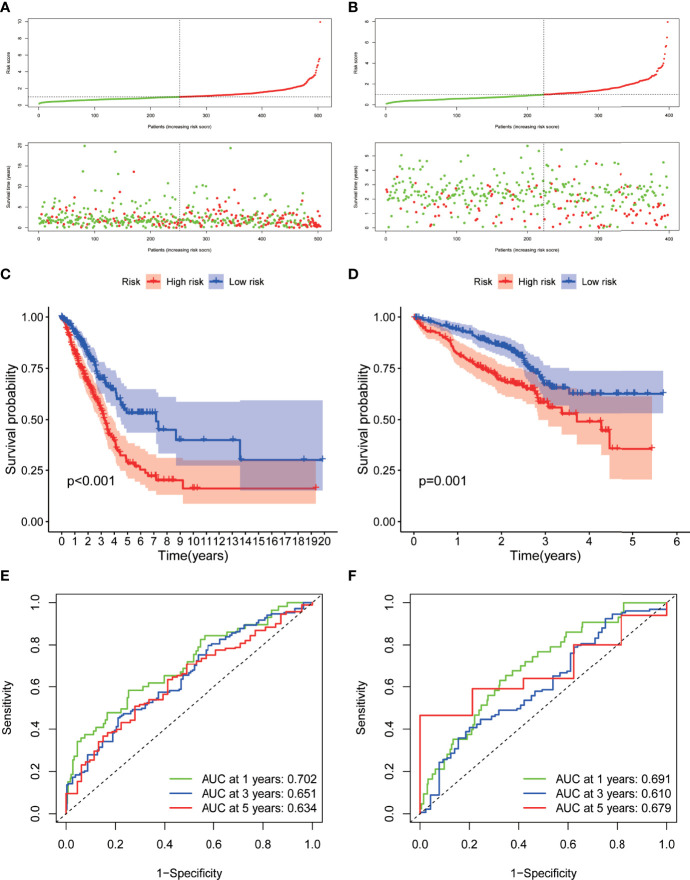
Development and validation of the hub-DRG-based prognostic model (RiskScore) A-B. Risk scores of the DRGs in TCGA cohort **(A)** and GSE72094 cohort **(B)** were calculated, and the patients were divided into high-risk group or low-risk group using the median risk score as the cutoff level. Upper panel: patient survival status and time distributed by the risk score. Bottom panel: risk score curves of the DRGs. **(C, D)**. Differences in overall survival between patients with high RiskScore and low RiskScore in TCGA-LUAD cohort **(C)** and GSE72094 cohort **(D)** were assessed using the log-rank test, as shown in the Kaplan–Meier plot. **(E, F)**. ROC plot showing the prognostic performance of the nomogram demonstrated by the time-dependent ROC curve for predicting the 1-, 3‐, and 5‐year survival rates in TCGA-LUAD training cohort **(D)** and GSE72094 validation cohort.

A prognostic model (RiskScore) integrating the expression level of the nine hub DRGs and their lambda value from Lasso analysis as coefficients was formulated as riskScore = SFTPB* -0.04820129 + WFDC2* -0.08632731 + HLA-DPA1* 0.22186232 + TIMP1* 0.26765595 + MS4A7*0.25567211 + HLA-DQA1*-0.15251071 + VCAN*0.12144478 + KRT8*0.26015183 + FABP5*0.22527365. Patients from TCGA cohort were categorized as high-risk group and low-risk group using the median value of RiskScore as the cutoff point (**Figure 6A**). The log-rank test showed that patients with low RiskScore had significantly better overall survival in TCGA-LUAD dataset (p < 0.001) (**Figure 6C**). To demonstrate the predicting power of RiskScore in an independent cohort, we evaluated the survival relevance of RiskScore in the GSE72094 dataset as a validation cohort. We obtained the risk score for each patients using the RiskScore model, based on which patients were categorized as high-risk group and low-risk group with the median value of risk score as the cutoff point. The high-risk group demonstrated significantly worse overall survival as compared to the low-risk group (p = 0.001) (**Figures 6B, D**). Time-dependent ROC curve analysis revealed impressive predictive power of the prognostic model in TCGA dataset [area under the curve (AUC) for prediction of 1-, 3-, and 5-year overall survival rates: 0.702, 0.651, and 0.632, respectively) (**Figure 6E**). The predictive ability of the prognostic model was also evaluated in an independent cohort (GSE72094), which showed comparable efficiency in predicting the overall survival rates at years 1, 3, and 5 years (AUC: 0.691, 0.610, and 0.679, respectively) (**Figure 6F**).

### Construction and Validation of Prognostic Nomogram Integrating RiskScore and Clinicopathological Parameters

**Figure 7:**
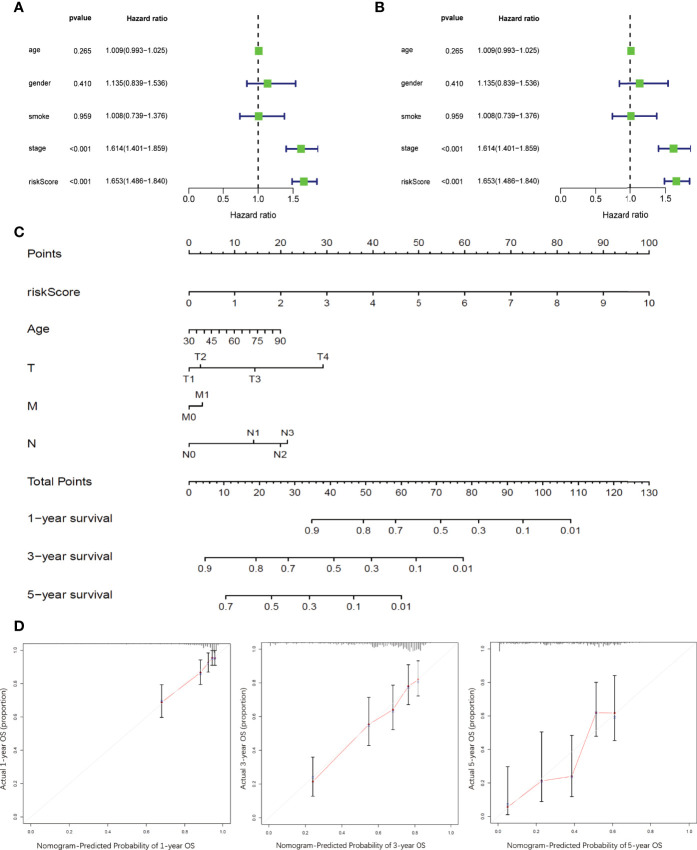
Development and validation of prognostic nomogram integrating RiskScore and conventional prognostic markers **(A, B)**. Forest plots showing the impact on overall survival of different risk factors as inferred by univariate analysis **(A)** and multivariate analysis **(B)**. **(C)** Nomogram model to predict the prognosis of LUAD patients based on TCGA training cohort. Age, sex, smoking history, tumor stages, and RiskScore inferred by hub DRGs. **(D)** Calibration plots of the prognostic nomogram for predicting overall survival at 1, 3, and 5 years in the TCGA.

Univariate and multivariate Cox survival analyses were conducted to compare the prognostic value of RiskScore with that of the other clinicopathological variables such as age, sex, smoking history, and TNM stage (**Figures 6A, B**). Only TNM stage and RiskScore showed significant prognostic relevance in both univariate and multivariate survival analyses (p < 0.001) (**Figures 7A, B**); the hazard ratios (95% confidential intervals) in multivariate analysis were 1.54 (1.33–1.783) and 1.649 (1.466–1.855), respectively.

A prognostic nomogram integrating RiskScore and clinicopathological variables such as age and TNM stage was developed based on TCGA-LUAD dataset to better predict the prognosis of LUAD patients (**Figure 7C**). Survival statuses at 1, 3, and 5 years were applied as parameters of clinical outcome. The calibration plots showed excellent agreement between the OS predictions and the actual observations of the 0.5-, 1-, and 3-year survival rates in TCGA cohort (**Figure 7D**).

### WFDC2 Promoted the Proliferation, Migration, and Invasion and Decreased Apoptosis of A549 Cells

**Figure 8:**
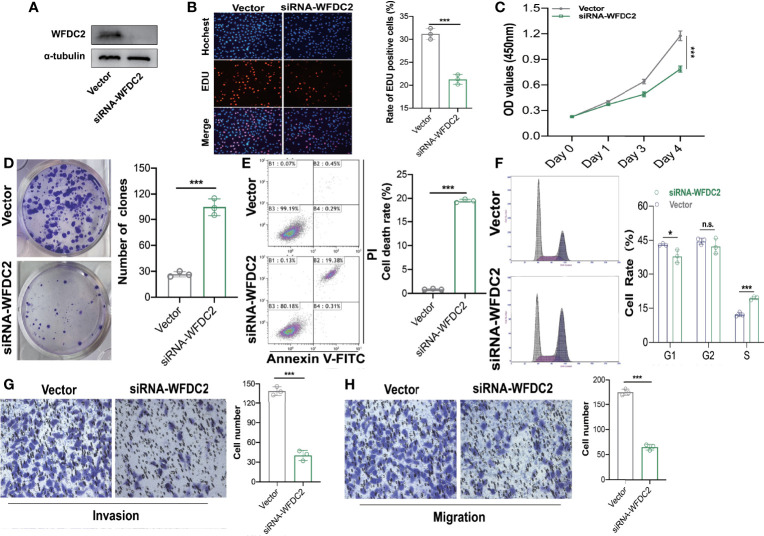
WFDC2 downregulation inhibited proliferation, migration, and invasion while enhanced apoptosis in A549 cells. **(A)** Expressions of WFDC2 inA549 cells transfected with or without siWFDC2 were detected by Western blot. **(B)** EdU assay was performed to detect the proliferation of A549 cells transfected with or without siWFDC2. **(C)** CCK-8 assay was performed to detect the proliferation of A549 cells transfected with or without siWFDC2. **(D)** Colony formation assay was performed to detect the proliferation of A549 cells transfected with or without siWFDC2. **(E)** Cell apoptosis of A549 cells transfected with or without si-WFDC2 was examined by Annexin V-FITC/PI-labeled flow cytometry. **(E)** Representative cell-cycle distribution profiles of WFDC2 inhibited 549 cells. **(F)**Transwell assay was performed to detect the migration and invasion of A549 cells transfected with or without si-WFDC2. n = 3, *p < 0.05,**p < 0.01,***p < 0.001.

WFDC2, which was mainly expressed in epithelial cells and served as an immunity-related gene, was most significantly differentially expressed in our own RNA-seq database of 19 LUAD patients (**Supplementary Figure 2**, **Supplementary Table 1**). Therefore, WFDC2 was chosen for further verification *in vitro*. To clarify the functions of WFDC2 in lung cancer, the expressions of WFDC2 were silenced in A549 cells (**Figure 8A**). WFDC2 knockdown significantly reduced EdU-positive cells (**Figure 8B**). The results of CCK-8 assay also showed that the proliferation of A549 cells was decreased when WFDC2 was silenced (**Figure 8C**). Meanwhile, WFDC2 depletion repressed the colony numbers of A549 cells (**Figure 8D**). These results indicated that knockdown of WFDC2 inhibited the proliferation of A549 cells. Then we conducted flow cytometry to examine the effects of WFDC2 on the apoptosis of A549 cells. Annexin V-FITC and PI staining revealed that the number of apoptotic cells was obviously increased when WFDC2 was silenced in A549 cells (**Figure 8E**). For cell-cycle analysis, we observed that silence of WFDC2 significantly increased the fraction of cells in the S phase while it decreased the fraction of cells in the G1 phase, which indicated that the cell proliferation of A549 cells was reduced (**Figure 8F**). Finally, Transwell assay indicated that WFDC2 inhibition also decreased the numbers of migrated (**Figure 8G**) and invaded A549 (**Figure 8H**) cells which indicated that WFDC2 inhibition attenuated the migration and invasion of A549 cells (Fig.2C). All these results indicated that WFDC2 acted as a pro-oncogenic regulator in lung cancer.

## Discussion

Utilizing the scRNA sequencing dataset, we explored intratumor heterogeneity by identifying cellular components of different origins and their differentiation status within the tumor mass, and their corresponding feature genes (DRGs). DRGs are highly correlated with pathways of immune regulation, whose expression patterns can classify LUAD into four groups with a distinct immune microenvironment and clinical outcomes. DRGs with independent prognostic significance were selected as hub genes for the construction of a prognostic model (RiskScore), which efficiently stratified LUAD patients with distinct prognosis. A nomogram integrating RiskScore and clinicopathological features was developed, which demonstrated impressive ability for prognostic prediction in LUAD.

Lung adenocarcinoma is a highly heterogenous entity in terms of genomic alteration, composition of intratumor cells, and tumor cell plasticity (4). While most previous studies have investigated the genomic features and their clinical significance (4, 39), only a few studies have tried to explore LUAD from the perspective of cellular heterogeneity. The rapid advances in single-cell sequencing technology and the corresponding analytic strategies have facilitated in-depth research on intratumor heterogeneity of LUAD. Different from the previous *in-silico* studies based on expression data of bulk tumor, where information of intratumor heterogeneity was lost during the analysis, scRNA seq data facilitate the evaluation of each single cell within the same tumor (40, 41). In the present study, we successfully identified different cellular clusters within tumor mass based on scRNA sequencing analysis. We anticipated that the distinct expression pattern of different cellular clusters within the same tumor mass can recapitulate the expression pattern of each bulk tumor. As shown by our findings, feature genes of different cellular clusters efficiently categorized tumors into different groups with distinct clinicopathological features and prognosis.

Feature genes of different clusters as identified by scRNA sequencing analysis were mostly associated with immune regulation pathways. Of note, LUAD classification based on the expression pattern of these feature genes also demonstrated distinct immune features across tumors of different groups. These findings indicated the intricate involvement of the immune context in the clustering of intratumor cells, which is either because of the different immune composition within different clusters or due to the different immune modeling effects of different cellular clusters. Tumors of cluster 3 were found to be associated with an immune-suppressive phenotype (a lower level of immune active composition and a higher level of immune-suppressive component) and worst clinical outcome. LUAD is known as an immune inflammatory cancer type with relatively higher infiltration of immune cells and a relatively favorable response to immunotherapy. Studies have shown that the composition and functional status of the immune population within the tumor mass play a key role in the development and progression of lung cancer (42). The immune context was found to be a major determinant of tumor characteristics in NSCLC and closely associated with clinical outcomes (43). Largely consistent with the published literature, we also found a significant association of immune cell infiltration and expression of immune-associated genes with prognosis in LUAD. As the major effector during immunotherapy, the immune composition is widely accepted to have a significant impact on the therapeutic efficacy of immune checkpoint inhibitors (44, 45). Although NSCLC is one of the cancer types that show the best response to immunotherapy, only a small fraction of patients actually benefit from the treatment (46). Patient classification based on cellular composition as applied in our study may offer a new strategy to identify patients who may benefit from immunotherapy.

Prognostic prediction is an essential part of clinical management of malignant disease, which can guide risk stratification and treatment decision-making (47). TNM staging is the most widely used approach for risk stratification of cancer patients in clinical settings; however, this approach does not accurately predict the prognosis of LUAD patients (48). Thus, development of strategies for a more precise prognosis prediction of LUAD is a key imperative. Plenty of prognostic signatures have been reported in published studies, whose prognostic genes were basically identified through differential expression analysis of bulk expression data and functional annotation of differentially expressed genes (49–51). A prognostic signature derived from bulk expression analysis tends to miss critical information such as the prognostic significance of different cellular components and the cellular origin of the prognostic genes. Our study sought to develop a prognostic model with feature genes derived from trajectory analysis of scRNA sequencing data. Among the finally selected hub genes with prognostic significance, six genes were mainly expressed by epithelial cells or stem cells, and the other three genes were highly expressed in immune cells, indicating that the immune population has an equally important impact on clinical outcomes. The prognostic model based on the hub genes outperformed all the clinicopathological parameters (including TNM staging) in predicting the clinical outcomes of LUAD patients. Our findings suggest that prognostic signature-derived feature genes of different cellular clusters within tumor mass can be a promising predictor for risk stratification of LUAD. To maximize the prognostic prediction ability, a strategy of integrating multiple parameters in a nomogram for risk stratification has been widely applied in research (52, 53). We developed a nomogram incorporating RiskScore (prognostic model based on hub gene expression) and clinicopathological features, which efficiently predicted both short-term and long-term survival of patients with LUAD. The concomitant use of RiskScore with conventional parameters can efficiently improve the accuracy of prognostic prediction in LUAD.

A total of nine hub genes for prognostic model construction were identified in our study. Three genes (*SFTPB*, *WFDC2*, and *HLA-DQA1*) were associated with reduced prognostic risk, while the other six genes (*HLA-DPA1*, *TIMP1*, *MS4A7*, *VCAN*, *KRT8*, and *FABP5*) were predictors of unfavorable survival. Consistently, in previous studies, a high expression of *TIMP1* (54, 55), *VCAN*, *KRT8* (56–58), and *FABP5* (59) and a low expression of *SFTPB* (60, 61), *WFDC2* (62, 63), and *HLA-DQA1* (64, 65) were associated with poor prognosis in multiple cancer types. However, a reduced expression of *HLA-DPA1* (64, 65) and *MS4A7* (66) was reported to be associated with disease progression and unfavorable survival, which differed from the findings in the studies. Especially, both *HLA-DPA1* and *HLA-DQA1* were reported to be immune genes associated with antigen presentation, whose reduction indicated an immune-suppressive microenvironment and aggressive disease (64, 65). The biological functions and prognostic role of all these hub genes identified in our study need to be validated and investigated in further studies.

In this study, we validated that WFDC2 was significantly upregulated in LUAD tissues in our own RNA-seq database among these nine hub genes. Then we conducted further *in vitro* investigations and demonstrated that WFDC2 might serve as a pro-oncogene to promote cell proliferation, migration, and invasion and inhibit apoptosis in A549 cells. Generally, WFDC2 is a secretory protein that could be detected in the serum and upregulated in various cancers including ovarian, endometrial, and breast cancer (67). WFDC2 has been shown to play vital roles in tumorigenesis, chemoresistance, and tumor metastasis and was confirmed to promote tube formation and enhance angiogenesis through STAT3 signaling (PMID: 32444701). Moreover, knockdown of WFDC2 decreased matrix metalloproteinase-2 (MMP-2) expression, both *in vitro* and *in vivo*, which indicated that WFDC2 may also contribute to cancer metastasis. Chen et al. discovered that the cell invasion induced by WFDC2 can be reversed by the inhibitor of P13K/AKT signaling (67), implying that WFDC2 promoted cancer invasion by activating the P13K/AKT signaling pathway. Interestingly, the serum level of WFDC2 was inversely correlated with cytotoxic T-cell infiltration, which indicated that WFDC2 might suppress proper T-cell trafficking and alter immunogenic responses in cancers (68). These results indicate that WFDC2 serves as a vital oncogene and is a potential therapeutic target for lung cancer.

Our study indicates that the cell differentiation trajectory-related genes are closely correlated with the immune contexture with tumor mass and efficiently predict prognosis in LUAD. Even with these interesting findings, several limitations of the current study need to be addressed. First of all, scRNA data were obtained from only three tumors, which might not be able to truly reflect the cellular composition in all the lung squamous cell carcinomas. Further study regarding single-cell sequences with a larger sample size is needed to confirm or expand our finding. Also, this was an *in-silico* study and the analysis was purely based on transcriptome data. The major findings were obtained from analysis of public datasets without further confirmation in data of our own cohort. Secondary, all datasets applied in our study mainly involved early-stage tumor samples. All the included cases underwent surgical treatment. The generalizability of our findings to advanced tumors needs to be evaluated in a future study. Thirdly, although the model developed in our study classified tumors with distinct immune contexture, we failed to evaluate its capacity in predicting response to immunotherapy due to the unavailability of relevant data. Last but not least, the calculation of RiskScore is based on the expression level of all the hub genes. As the magnitude of expression data differs across different platforms, the generalized application of RiskScore in the clinical practice is restricted.

## Conclusions

To conclude, our study systematically evaluated the tumor heterogeneity of LUAD and assessed its clinical relevance based on the *in-silico* analysis integrating scRNA sequencing data and RNA sequencing data of bulk tumors. Genes associated with cellular differentiation trajectory are closely associated with immune contexture and can predict prognosis of patients with LUAD.

## Data Availability Statement

The raw data supporting the conclusions of this article will be made available by the authors, without undue reservation.

## Author Contributions

YL, XD, LC, and HW designed the study and contributed to the study materials and consumables. YL, XD, and JQ conducted the study. YL, GD, and WX collected data. YL, XD, and ZL performed the statistical analyses and interpreted the data. YL, XD, LC, and HW wrote the manuscript. All authors contributed to the manuscript and approved the manuscript.

## Funding

This work was supported by the National Natural Science Foundation of China (Grant No. 81972175), Major Program of Science and Technology Foundation of Jiangsu Province (No. BE2018746), and the Program of Jiangsu Medical Innovation Team (No. CXTDA2017006).

## Conflict of Interest

The authors declare that the research was conducted in the absence of any commercial or financial relationships that could be construed as a potential conflict of interest.

## Publisher’s Note

All claims expressed in this article are solely those of the authors and do not necessarily represent those of their affiliated organizations, or those of the publisher, the editors and the reviewers. Any product that may be evaluated in this article, or claim that may be made by its manufacturer, is not guaranteed or endorsed by the publisher.
