# Supplementary material for: Cell Trajectory-Related Genes of Lung Adenocarcinoma Predict Tumor Immune Microenvironment and Prognosis of Patients
Source: Front Oncol. 2022 Jul 18;12:911401. doi: 10.3389/fonc.2022.911401 (PMC9339705; doi:10.3389/fonc.2022.911401)
Supplement: Supplementary file 1 [file DataSheet_1.pdf]

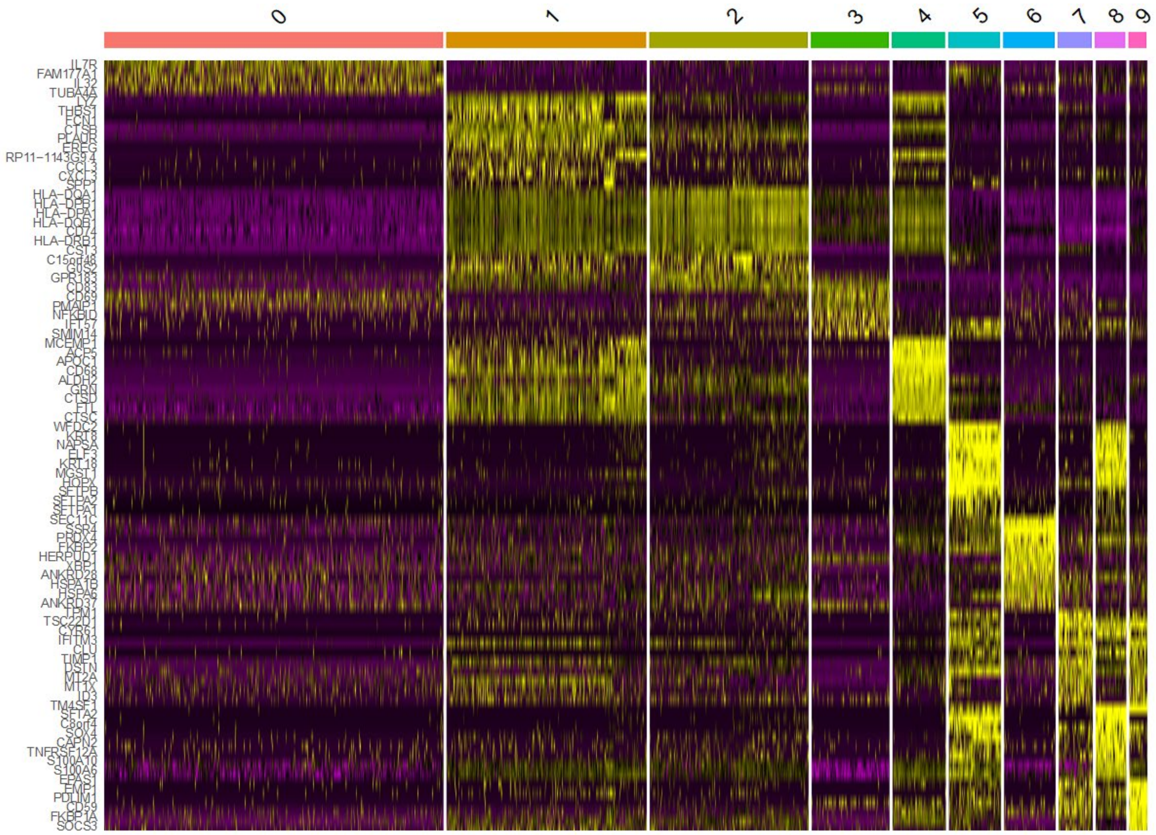

Supplementary Figure 1. Heatmap demonstrating the differentially expressed genes across the 10 clusters. The expressions of top 20 marker genes from each cluster are shown. The colors from purple to yellow indicate the gene expression levels from low to high.

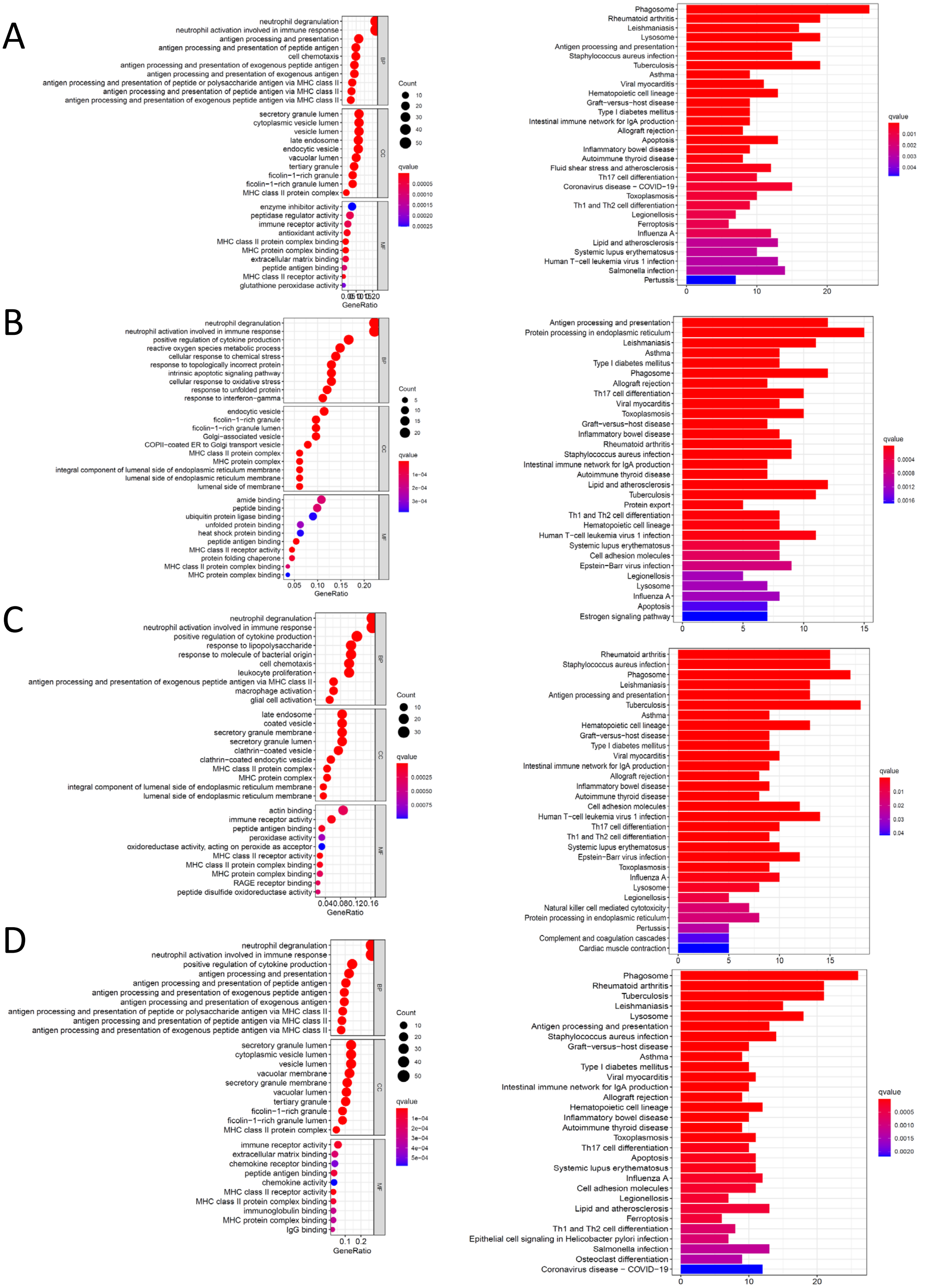

Supplementary Figure 2. Significant pathways from GO and KEGG analysis for marker DRGs of cells on different branches are shown respectively. A: GO and KEGG for subset I; B for subset III; C for subset IV; D for subset V.

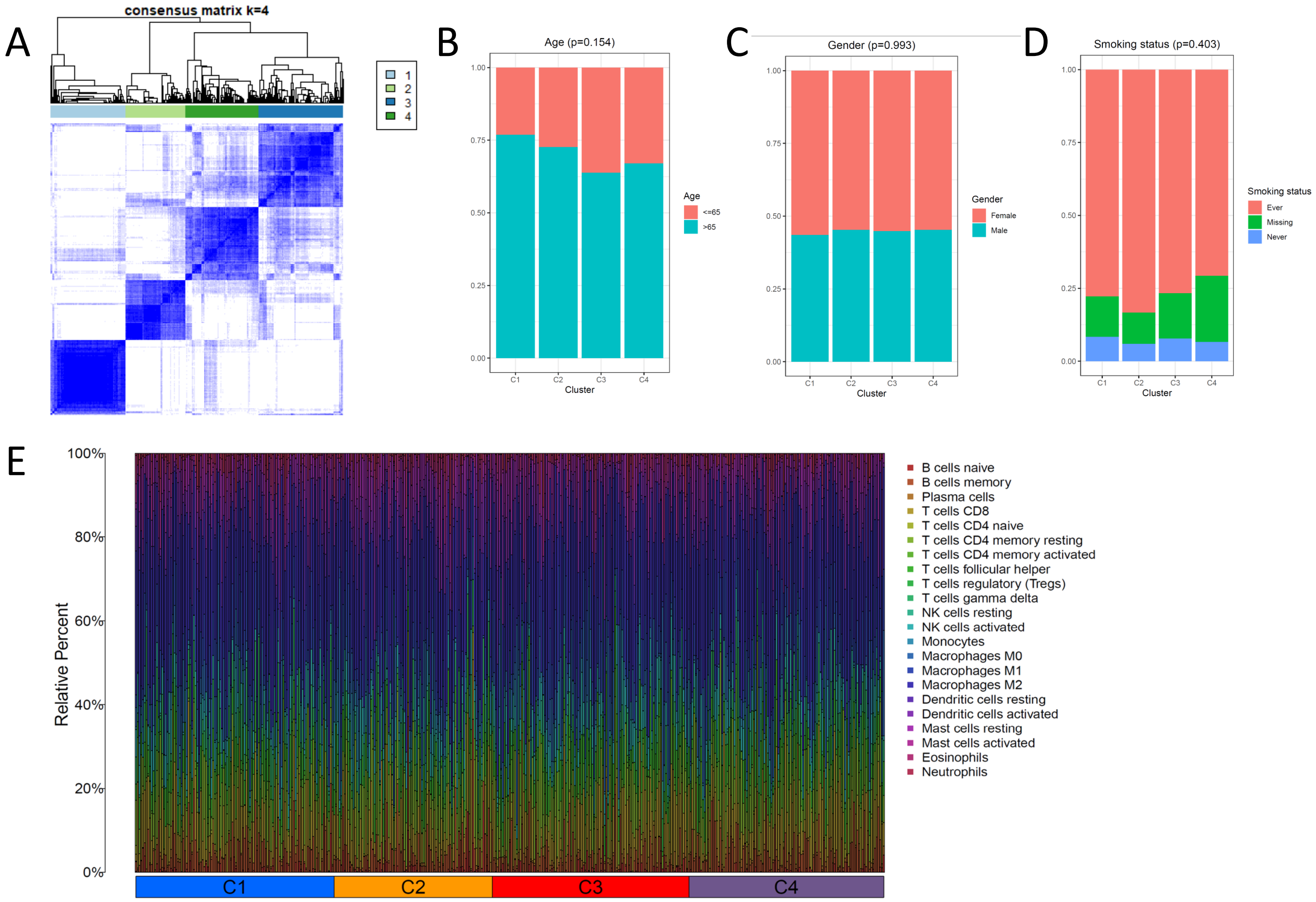

Supplementary Figure 3. DRGs-based classification of LUAD patients and their corresponding clinicopathological and immune infiltration features. A. Consensus clustering matrix for k = 4, which was the optimal cluster number in the issue-bulk RNA-seq data of GSE72094 cohort. B-D: Comparisons of the clinicopathological variables including age (B), sex (C), smoking status (D). E: Composition of 22 different subtypes of immune cells (inferred by Cibersort) in each individual sample of four different groups are shown in the percentage bar plot.

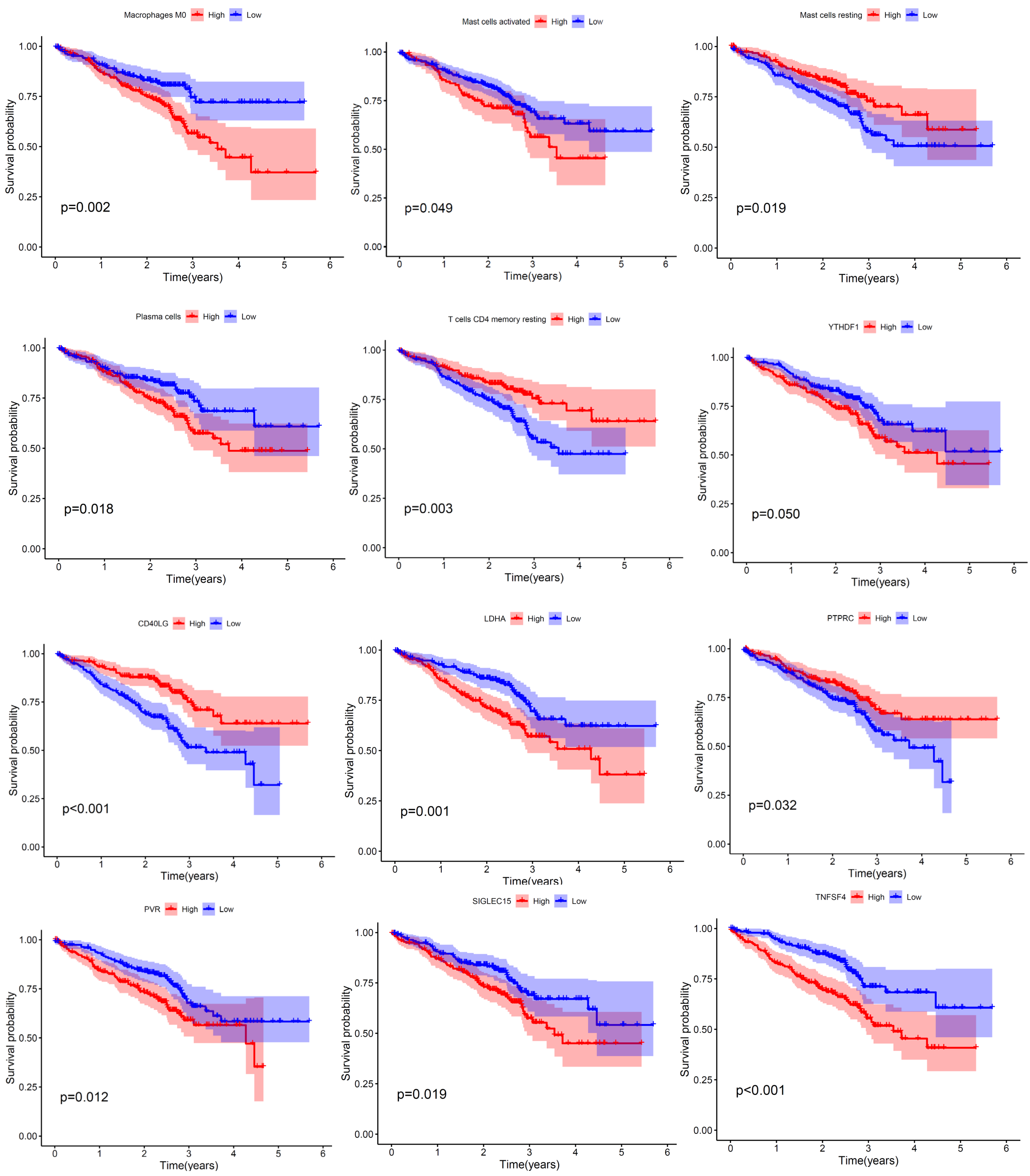

Supplementary Figure 4. Survival impact of different immune populations and immune associated genes in LUAD

Kaplan-Meier plots of overall survival difference between tumors with high and low level of different immune cells infiltration or immune relating genes expression

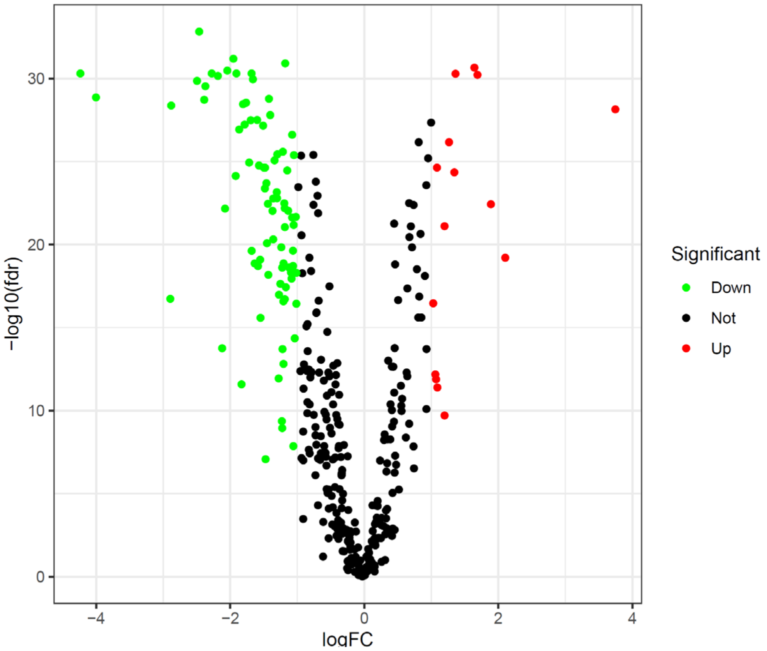

Supplementary Figure 5. Volcano plot showing the differentially expressed DRGs between malignant cells of LUAD and normal cells derived from adjacent normal tissue. Genes with significantly high expression in tumor cells are denoted as red dots, while genes with significantly high expression in normal cells are represented by green dots; genes demonstrating no significant differential expression are shown as black dots.

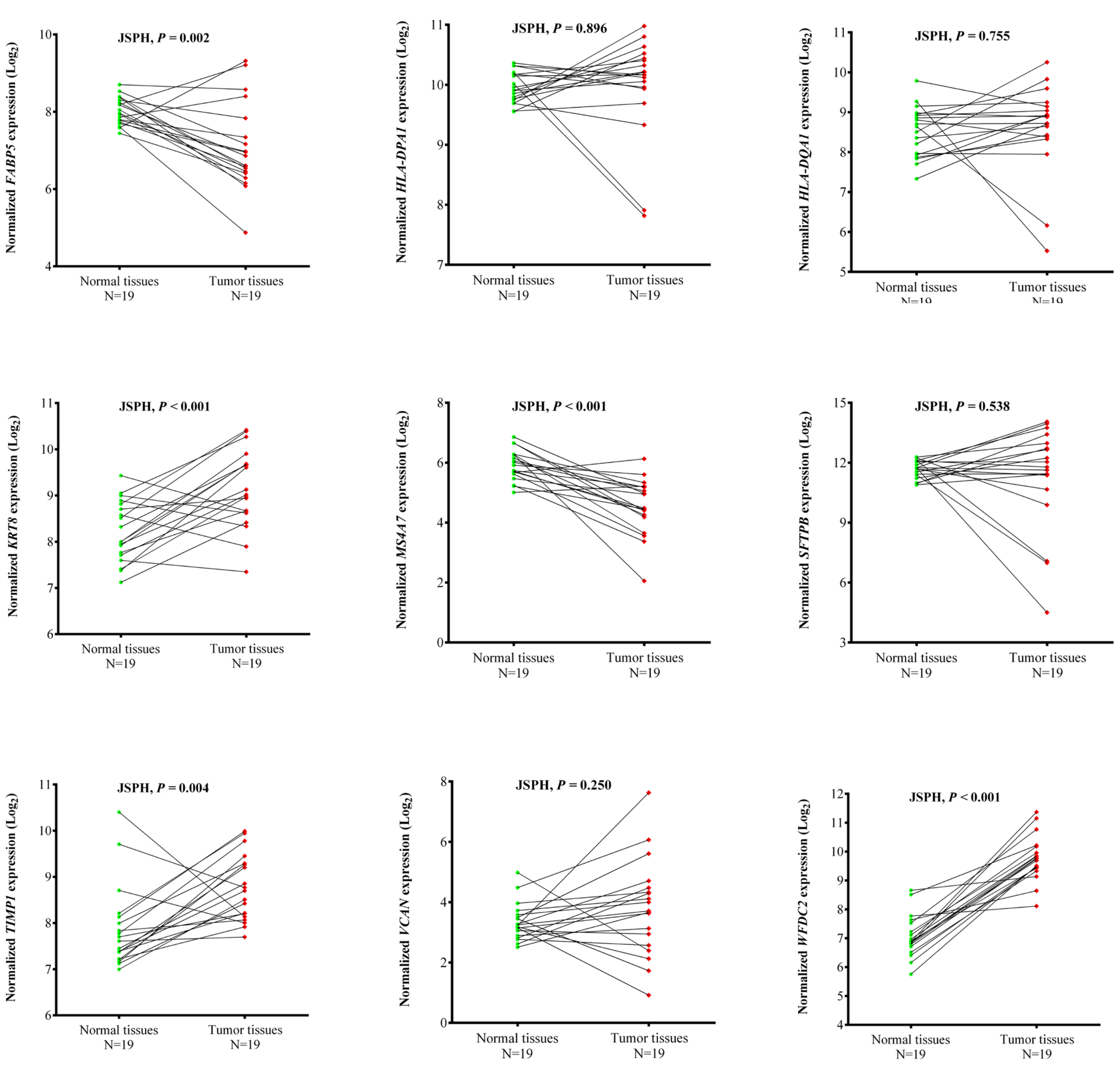

Supplementary Figure 6. Pairwise comparison of the expression level of the 9 hub genes between tumor tissue and adjacent normal tissue

Pairwise-T test was performed to evaluated the differential expression status of each gene between tumor tissue and adjacent normal tissue
